# LacaScore: a novel plasma sample quality control tool based on ascorbic acid and lactic acid levels

**DOI:** 10.1007/s11306-016-1038-1

**Published:** 2016-04-27

**Authors:** Jean-Pierre Trezzi, Alexandre Bulla, Camille Bellora, Michael Rose, Pierre Lescuyer, Michael Kiehntopf, Karsten Hiller, Fay Betsou

**Affiliations:** Integrated Biobank of Luxembourg, 6 rue Ernest Barble, 1210 Luxembourg, Luxembourg; Luxembourg Centre for Systems Biomedicine, 7, Avenue des Hauts-Fourneaux, 4362 Esch-Belval, Luxembourg; Sérothèque Centrale, Département de Médecine Génétique et de Laboratoire, Hôpitaux Universitaires de Geneve, 1211 Geneve 14, Switzerland; Institute of Clinical Chemistry and Laboratory Diagnostics and Integrierte BioBank Jena (IBBJ), Jena University Hospital, Erlanger Allee 101, 07740 Jena, Germany; International Society for Biological and Environmental Repositories (ISBER) Biospecimen Science Working Group, Vancouver, Canada

**Keywords:** GC/MS, Metabolomics, Plasma processing, Pre-analytics, Biobank, Quality control

## Abstract

**Introduction:**

Metabolome analysis is complicated by the continuous dynamic changes of metabolites in vivo and ex vivo. One of the main challenges in metabolomics is the robustness and reproducibility of results, partially driven by pre-analytical variations.

**Objectives:**

The objective of this study was to analyse the impact of pre-centrifugation time and temperature, and to determine a quality control marker in plasma samples.

**Methods:**

Plasma metabolites were measured by gas chromatography-mass spectrometry (GC–MS) and analysed with the MetaboliteDetector software. The metabolites, which were the most labile to pre-analytical variations, were further measured by enzymatic assays. A score was calculated for their use as quality control markers.

**Results:**

The pre-centrifugation temperature was shown to be critical in the stability of plasma samples and had a significant impact on metabolite concentration profiles. In contrast, pre-centrifugation delay had only a minor impact. Based on the results of this study, whole blood should be kept on wet ice and centrifuged within maximum 3 h as a prerequisite for preparing EDTA plasma samples fit for the purpose of metabolome analysis.

**Conclusions:**

We have established a novel blood sample quality control marker, the LacaScore, based on the ascorbic acid to lactic acid ratio in plasma, which can be used as an indicator of the blood pre-centrifugation conditions, and hence the suitability of the sample for metabolome analyses. This method can be applied in research institutes and biobanks, enabling assessment of the quality of their plasma sample collections.

**Electronic supplementary material:**

The online version of this article (doi:10.1007/s11306-016-1038-1) contains supplementary material, which is available to authorized users.

## Introduction

Sample collection and processing for research are not always performed in a strictly standardized manner. Biological samples are sensitive to molecular and/or environmental stress and are often not handled with the optimal precautions, generating an urgent need for validated sample processing methods and quality control, especially in the scope of “-omics” analyses (Balasubramanian et al. 2010). In order to support the need for evidence-based protocols, a series of sample processing method validation studies have been published (Ammerlaan et al. 2014a, b). In addition, biobanks and research institutes are facing the problem that quality and the fitness-for-purpose of a given legacy sample is often not known, resulting in research projects being highly error-prone (Moore et al. 2011). For prospective collections, the workflow should thus be rigorously defined and validated for fitness-for-purpose. For legacy collections of unknown pre-analytical history, quality controls must be developed and applied.

Metabolome analysis is complicated by continuous and dynamic changes of metabolite concentrations. Studying metabolites, small intermediates of thousands of enzyme-catalysed biochemical reactions, is a critical part of the field of metabolomics. Metabolites connect different intra- and extracellular metabolic pathways, and ensure homeostasis (Wishart et al. 2007). The metabolome, the total set of metabolites, is considered to be a final phenotype, based on the combinatorial effects of the genome, transcriptome, proteome and environment. Metabolome analyses may thus provide the most sensitive approach for detecting cellular and environmental changes. Many metabolites have a very limited half-life as they are constantly produced and degraded by biochemical reactions (Villas-Boas et al. 2005). This property complicates metabolite analyses in a time-limiting manner, highlighting the need for rapid sample processing, as well as rapid quenching and accurate metabolite measurement methods.

The plasma metabolome is subject to in vivo pre-analytical variations, related to race (Gavaghan McKee et al. 2006), sex and age (Kochhar et al. 2006), intestinal flora (Phipps et al. 1998), circadian rhythms and the oestrous cycle (Bollard et al. 2001), as well as diet and lifestyle (Lenz et al. 2004). The impact of in vitro pre-analytical variations on metabolomics data has recently been investigated on nuclear magnetic resonance (NMR) and liquid chromatography–mass spectrometry (LC–MS) analytical platforms (Bernini et al. 2011; Fliniaux et al. 2011; Kamlage et al. 2014; Yin et al. 2013).

In this study, we analysed the impact of pre-analytical variations, i.e. pre-centrifugation time and temperature, on the plasma metabolome. In a first phase, sensitive non-targeted GC–MS analysis was applied as a discovery tool. In a second phase, specific targeted enzymatic assays for the metabolites of interest were applied. Based on these findings, we established a novel sample quality control tool for the “diagnosis” of the quality of a given plasma sample with respect to pre-centrifugation conditions.

## Materials and methods

### Study design

Initially, the effects of pre-centrifugation storage temperature (4 °C) versus room temperature (RT; 18–23 °C), and pre-centrifugation delay (10, 30, and 60 min) on metabolite concentrations in blood samples from three healthy volunteers were determined using GC–MS.

Based on the outcome of this, a second part of the study was initiated, evaluating lactic acid and ascorbic acid concentrations using enzymatic assays in plasma samples from a second cohort of ten healthy volunteers collected with a range of pre-centrifugation delays (30 min, 3 h, or 23 h) at 4 °C and RT. For this,10 mL blood samples from ten healthy donors were collected in K2EDTA collection tubes (BD #367525) and stored at RT or on ice for pre-centrifugation delays of 30 min, 3 h, or 23 h.

These data were used to determine a “LacaScore” and a LacaScore threshold, which was established using ROC analysis. This threshold and the LacaScore’s performance were validated in plasma samples from three independent collections from three different institutes: (1) samples from 25 donors from different, previously constituted, IBBL collections, with a range of blinded pre-centrifugation delays at RT, (2) samples from 16 donors collected and processed at the Geneva University Hospitals Biobank, with a range of pre-centrifugation delays (30 min, 3 h, or 23 h) at 4 °C and RT, (3) samples from 20 donors, collected and processed at the Jena University Hospital, with a range of pre-centrifugation delays (30 min, 1, 2, 4 h) at RT. A final analysis of “extreme” conditions was performed in blood samples from a cohort of five healthy donors collected immediately post-exercise with a range of pre-centrifugation delays (1, 2, 3, 4, or 6 h) at 4 °C and RT.

### Plasma processing

All blood sample donors have signed informed consent forms and the study was approved by ethics committees (CNER approval #201107/02 for IBBL, CEREH approval #13-203 for Geneva University Hospital and #3921-11/13 for Jena University Hospital).

For the GC–MS analysis, 10 mL blood samples were collected from three healthy volunteers in K2EDTA 10 mL tubes (BD #367525) and centrifuged for 10 min at 4 °C, 2000×*g*, brake five (soft). The supernatant was then aliquoted (10 × 200 µl aliquots per condition) and stored at −80 °C until metabolite extraction. The study was divided into two parts: analysis of the impact of the (1) processing temperature and (2) processing time on metabolomics data. For (1), two processing temperatures were tested: wet ice (4 °C) and RT (18–23 °C). For (2), pre-centrifugation delays of 10, 30, and 60 min were tested.

For the targeted enzymatic assays, 10 mL blood samples were collected in K2EDTA 10 mL tubes (BD #367525) and centrifuged for 20 min at RT, 2000×*g*, brake five (soft). The supernatant was then aliquoted and stored at −80 °C until testing (IBBL and Geneva University Hospital). Moreover, 2.7 mL blood samples were collected in K3EDTA tubes (Sarstedt #05.1167.001) and centrifuged at 2500×*g* for 10 min at 20 °C. The supernatant was then aliquoted and stored at −80 °C until testing (Jena University Hospital).

### Metabolite extraction

Each plasma sample was extracted in triplicate. In addition, plasma pools were produced by mixing an equal amount of each sample. For each replicate, 5 µL of plasma sample were mixed with 45 µL ice–cold methanol–water mix (MeOH/H_2_O; 8/1; v/v) and vortexed on a shaking device for 5 min at 4 °C. The mix was directly centrifuged at 16,000×*g* for 5 min at 4 °C (Eppendorf 5415R), then 30 µL supernatant was transferred into GC glass vials and completely dried by a refrigerated CentriVap Concentrator (Labconco) at −4 °C for 40 min. To avoid condensation on the vials, the CentriVap Concentrator was allowed to warm to RT for 30 min prior to taking out the vials.

### Gas chromatography–mass spectrometry (GC–MS)

GC–MS measurements were performed on an Agilent 6890 gas chromatograph equipped with a DB-35MS capillary column. The GC is coupled to an Agilent 5975C MS equipped with an electron impact (EI) ionization source operating at 70 eV. The mass spectrometer source was heated to 230 °C and the quadrupole to 150 °C.

Metabolite derivatization was performed using a Gerstel Multipurpose Sampler. Dried metabolite extracts in GC glass vials were mixed with 15 µL of 2 % methoxyamine hydrochloride in pyridine (MOX, Sigma Aldrich) and incubated at 40 °C. After 30 min, 15 µL of MSTFA (2,2,2-trifluoro-*N*-methyl-*N*-trimethylsilyl-acetamide, Machery Nagel) was added and the mixture was further incubated for 30 min at 40 °C.

The detector was operated in scan mode and 1 µL of derivatized sample was injected in the GC sample inlet. The injection was set to splitless mode and helium was used as the carrier gas (flow rate: 1 mL/min). The GC oven was maintained at 80 °C for 6 min, then increased to 300 °C (heating rate: 6 °C/min). Thereafter, the temperature was increased to 325 °C for 4 min (heating rate: 10 °C/min). The run of one sample took 1 h.

GC–MS raw data were calibrated using the MetaboliteDetector software (Hiller et al. 2009) retention index calibration option. Batch quantification (non-targeted analysis) was performed to obtain quantitative data for statistical analysis by multivariate ANOVA. The level of significance was set to *p* < 0.05. With our GC–MS instrumentation, we detected ascorbic acid levels, but not dehydroascorbic acid.

### Enzymatic assays

Ascorbic acid and lactic acid levels were quantified in the plasma by enzymatic assays (EnzyChrom Ascorbic Acid Assay Kit, BioAssay Systems, EASC-100; L-Lactate Assay Kit, Abcam, ab65331). All samples were measured in duplicate. With the enzymatic assay, we detected ascorbic acid levels, but not dehydroascorbic acid.

The manufacturer’s protocol was followed for lactic acid quantification, using 3 µL plasma samples. Colorimetric measurement was performed by a microplate reader at OD_450_.

The ascorbic acid quantification protocol was adapted to enable the measurement of low ascorbic acid concentrations. A total of 50 µL of plasma was mixed with 50 µL reaction mix and incubated for 10 min at RT in the dark. Fluorimetric measurements were performed by a microplate reader at λ_ex_ = 530 nm and λ_em_ = 585 nm.

These enzymatic assays were also performed using 25 independently-collected, plasma samples from IBBL collections with a range of blinded pre-centrifugation delay and temperature storage conditions, 94 plasma samples from Geneva University Hospitals Biobank collections and 80 plasma samples from Jena University Hospital. In addition, to determine whether our findings were robust against high lactic acid and ascorbic acid baseline levels, we sampled blood from five healthy donors, immediately after a half-marathon run (21 km). For each donor, blood samples were collected and stored on ice or at RT for 1, 2, 3, 4, or 6 h. One runner (D3) took 2 g ascorbic acid in the evening preceding the run. Due to the extreme lactic acid and ascorbic acid levels, the sample dilution factors in the assays were adapted. For lactic acid quantification, plasma was diluted 1:2 in assay buffer, and for ascorbic acid quantification, plasma was diluted 1:3 in milliQ water before quantification according to the recommendation of the manufacturers.

Lactic acid and ascorbic acid concentration values were used for LacaScore calculation: for each sample, the ascorbic acid level was divided by the corresponding lactic acid level and multiplied by 10^5^ (LacaScore = ascorbate [µM]/lactate [µM] × 10^5^).

### ROC analysis

**Fig.1:**
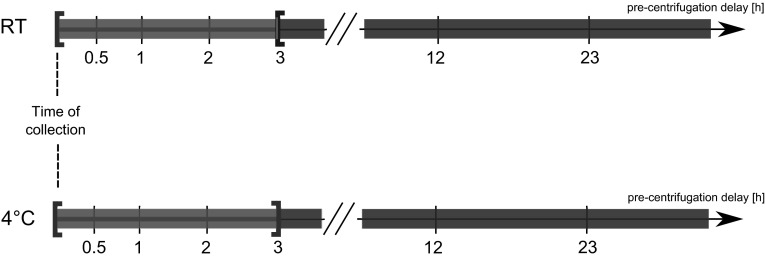
Representation of ranges of pre-analytical conditions, at RT and 4 °C, corresponding to “high pre-centrifugation quality” or “positive” samples (*green*) and “low pre-centrifugation quality” or “negative” samples (*red*) (Color figure online)

Based on the data of the 10 enzymatic assay donors, we established a LacaScore threshold by ROC analysis. As a decision threshold, plasma samples considered “high pre-centrifugation quality” (or “positive”) were qualified as those extracted from blood stored either at 4 °C for up to 3 h or at RT for up to 2 h before centrifugation (Fig. 1). The remaining plasma samples were qualified as “low pre-centrifugation quality” (or “negative”). The ROC analysis was performed in R 3.2.2 using the pROC package (Robin et al. 2011).

## Results

In this study, we aimed to identify markers for the assessment of the quality of plasma sample collections in biobanks and research institutes. We analysed the impact of two pre-analytical conditions on plasma sample metabolites: pre-centrifugation delay and temperature.

### Significant impact of pre-analytical factors on the plasma metabolome

In a first step, we compared the metabolomics signature of plasma samples obtained from three healthy donors. For each donor, we collected blood into two K2EDTA blood collection tubes which we immediately stored on wet ice or RT. For each condition, we stored the tubes for 10, 30 and 60 min before plasma processing, such delays being in accordance with previously published recommendations (Fliniaux et al. 2011; Bernini et al. 2011; Kamlage et al. 2014; Yin et al. 2013).

MANOVA analysis of GC–MS analysed samples revealed that for the 1 h time course applied in this experiment, the pre-centrifugation time had a minor impact whereas the storage temperature had a significant impact on the plasma metabolome. Only 3 out of 236 (~1.3 %) detected metabolites were significantly increased over the 1 h time course: RI 1035.67, RI 1614.13 and RI 1573.94 (*p* = 0.008, *p* = 0.033 and *p* = 0.049, respectively). These metabolites could not be identified by our in-house compound library and are therefore presented as their respective retention indices (RI).

In contrast to the pre-centrifugation delay, the storage temperature had a significant impact on the levels of 48 out of the 236 (20.3 %) detected metabolites (Table 1, supplementary Data 1). Three metabolites showed a false discovery rate (FDR) <0.05 after correction for multiple testing. Lactic acid levels were significantly higher in samples stored at RT compared to the wet ice stored samples (FDR = 0.001). Ascorbic acid and RI 1809.71 levels were significantly lower at RT compared to 4 °C (FDR = 0.003 and FDR = 0.004, respectively).

**Table 1:** The impact of storage temperature prior to centrifugation on the metabolic profile in plasma

Metabolite	RT^**a**^ (mean ± SD)	Ice^**a**^ (mean ± SD)	Fold change (RT vs. Ice)	*P* ^**^	Adjusted *p* (Benjamini & Hochberg)
Ascorbic acid	1.717 ± 1.043	16.572 ± 5.979	0.104	<0.001	0.003
Carbonic acid	1.445 ± 0.385	2.026 ± 0.209	0.713	0.003	0.155
RI 1809.71	3.877 ± 0.492	4.646 ± 0.383	0.835	<0.001	0.004
RI 1193.68	1.398 ± 0.098	1.604 ± 0.320	0.871	0.006	0.182
RI 1441.25	1.069 ± 0.080	1.133 ± 0.059	0.943	0.007	0.182
RI 1027.62	1.141 ± 0.255	1.097 ± 0.454	1.040	0.005	0.173
RI 1056.57	1.135 ± 0.800	1.081 ± 0.470	1.050	0.002	0.125
RI 3088.55	1.139 ± 0.148	1.084 ± 0.314	1.051	0.012	0.182
RI 1132.34	1.129 ± 0.048	1.056 ± 0.040	1.069	0.004	0.161
Lactic acid	1.649 ± 0.185	1.055 ± 0.141	1.563	<0.001	0.001

^a^Normalized peak intensities

^**^Computed by MANOVA, level of significance p < 0.05

Significant metabolic changes observed in concentrations of the 236 detected metabolites when comparing room temperature (RT) and ice-stored (Ice) blood samples for the 60 min time course. The top ten metabolites are shown. The complete list can be seen in Supplementary Data 1

### LacaScore for plasma quality assessment

Having established that lactic acid and ascorbic acid are the most temperature-sensitive metabolites, we then extended the pre-centrifugation time period beyond 60 min. Two further time points were chosen. The first, based on published recommendations for metabolomics (Yin et al. 2013; Bernini et al. 2011), was at 3 h, corresponding to a critical time for compatibility with analyses which are sensitive to pre-centrifugation conditions. The second was 23 h, chosen as a time point at which it is certain that the sample quality for sensitive analyses is compromised.

As GC–MS instruments are not routinely operated in biobanks and research institutes, we quantified lactic acid and ascorbic acid levels using largely accessible enzymatic assays. We calculated the ratio of ascorbic acid to lactic acid for each sample and multiplied this ratio by 10^5^, hereafter termed the “LacaScore”.

**Fig.2:**
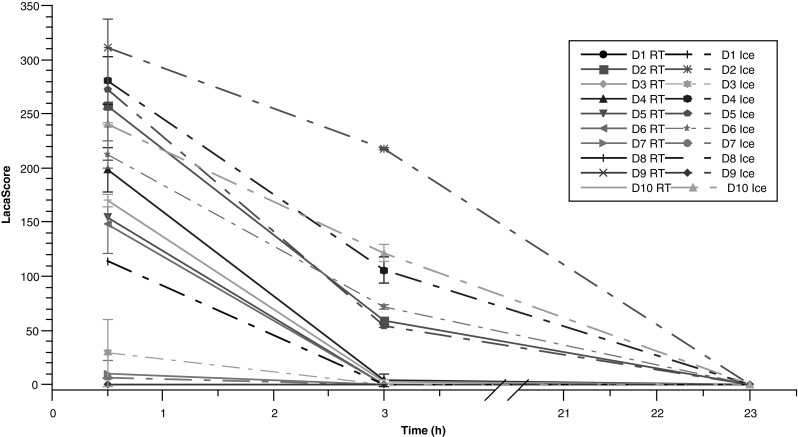
LacaScore showing significant differences due to pre-analytical conditions. The LacaScore is found to be higher in samples stored on ice (*dashed lines*) compared to samples stored at room temperature (solid lines). D1–D10 Donor 1-Donor10; *RT* room temperature storage (18–23 °C); *Ice* Ice storage (4 °C)

The LacaScore decreased over time between 30 min and 3 h at both 4 °C and RT (Fig. 2), due to a decrease of ascorbic acid and an increase of lactic acid levels (Supplementary Data 2). A significant difference of the LacaScore was observed between 4 °C and RT stored samples after a 30-min pre-centrifugation delay, validating our findings from the GC–MS study. All samples from donors #8 and #9 showed very low initial ascorbic acid levels and could not be quantified by the enzymatic assay (below the limit of quantification of the method). Therefore, the LacaScore for all these samples was set to 0.

### LacaScore threshold and validation in independent–datasets

**Fig.3:**
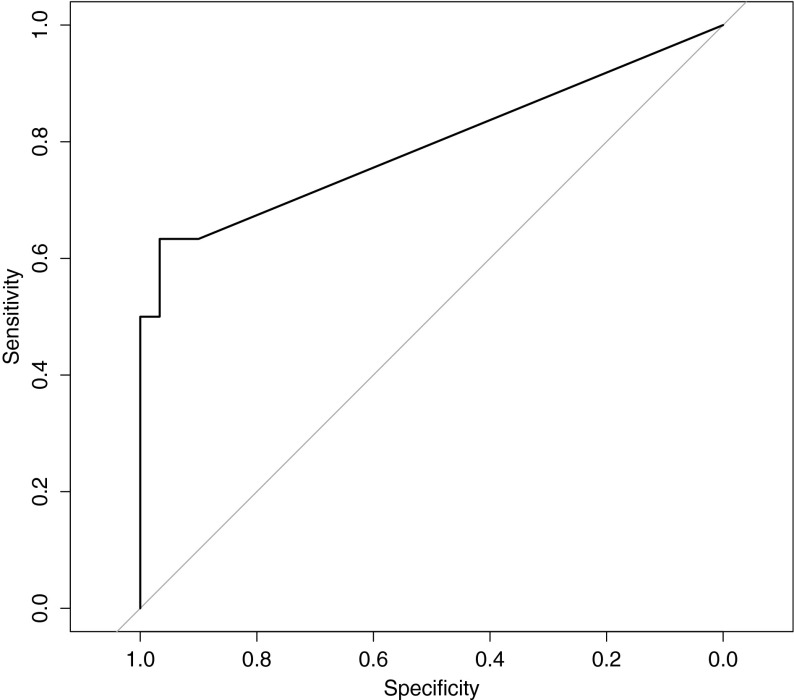
ROC curve for the determination of an optimal decision threshold (AUC = 0.794). The ROC analysis was performed on the LacaScores calculated on the enzymatic assay results based on 10 healthy donors. High pre-centrifugation quality samples were considered as “positive”. At a LacaScore threshold of 5.2, a specificity of 0.967 and a sensitivity of 0.633 were obtained

Based on the data from the ten donors we calculated a LacaScore diagnostic threshold. We qualified as “high pre-centrifugation quality” all samples stored either on ice for up to 3 h or at RT (18–23 °C) for less than 3 h. As “low pre-centrifugation quality”, we defined all samples stored longer than 3 h on ice, or 3 h or longer at RT. The ROC analysis based on the LacaScore data from the ten donors showed an optimal LacaScore threshold of 5.2 with specificity = 0.967 and sensitivity = 0.633 (Fig. 3).

To validate the LacaScore threshold, we used a blinded set of 25 plasma samples collected at RT with a range of pre-centrifugation delays. Based on the threshold of LacaScore 5.2, the diagnostic accuracy was 68 %. When we assigned samples with LacaScore between 5.2 and 52 to a category qualified as “indeterminate”, and included the samples of “indeterminate” quality status among the accurately diagnosed ones, the diagnostic accuracy increased to 88 % (Table 2), and this was selected as the final algorithm. With these settings, LacaScore <5.2 corresponds to EDTA plasma samples with pre-analytical quality equivalent to either <3 h RT or ≤3 h 4 °C pre-centrifugation delay, LacaScore >52 corresponds to EDTA plasma samples with pre-analytical quality equivalent to ≥3 h RT or >3 h 4 °C pre-centrifugation delay (Fig. 1), while 5.2 ≤ LacaScore ≤ 52 corresponds to indeterminate pre-analytical quality. Application of this algorithm to the two other plasma collections from Geneva University Hospitals and from Jena University Hospital gave diagnostic accuracies of 71 and 86 % respectively (Table 3). The detailed results from these collections are presented in Supplementary Data 3 and 4.

**Table 2:** Quality diagnosis based on the LacaScore determined from 25 independent biobank plasma samples with various pre-centrifugation delays at room temperature

Sample	Pre-centrifugation delay at RT (h)	Pre-centrifugation quality	LacaScore	Diagnosed pre-centrifugation quality^a^
S1	6:10	Low	0.000	Low
S2	7:36	Low	0.000	Low
S3	3:30	Low	0.000	Low
S4	4:52	Low	54.436	High
S5	2:30	High	11.475	Indeterminate
S6	5:35	Low	0.000	Low
S7	6:25	Low	51.388	Indeterminate
S8	3:47	Low	11.130	Indeterminate
S9	7:00	Low	0.000	Low
S10	6:00	Low	0.000	Low
S11	3:40	Low	0.000	Low
S12	5:55	Low	40.896	Indeterminate
S13	4:20	Low	0.000	Low
S14	2:54	High	137.956	High
S15	6:27	Low	0.000	Low
S16	1:50	High	618.312	High
S17	0:52	High	104.279	High
S18	0:52	High	0.000	Low
S19	2:42	High	236.286	High
S20	7:30	Low	34.784	Indeterminate
S21	7:40	Low	0.000	Low
S22	9:56	Low	0.000	Low
S23	3:22	Low	82.012	High
S24	2:16	High	190.559	High
S25	3:54	Low	9.303	Indeterminate

^a^LacaScore > 52: high pre-centrifugation quality; LacaScore < 5.2: low pre-centrifugation quality; 5.2 ≤ LacaScore ≤ 52: indeterminate quality; diagnostic accuracy: 88 % *RT* room temperature storage (18–23 °C)

**Table 3:** Quality diagnosis based on the LacaScore determined from 36 independent biobank plasma samples with various pre-centrifugation delays at room temperature or 4 °C

Temperature	Time (h)	Pre-centrifugation quality	LacaScore range [min–max]	Correctly diagnosed samples
Geneva University Hospital collection (n = 16)				
RT	0.5	High	[0–1487.41]	5 out of 16
RT	3	Low	[0–1311.70]	13 out of 15
RT	23	Low	[0–6.67]	16 out of 16
4 °C	0.5	High	[0–1314.18]	10 out of 16
4 °C	3	High	[0–547.76]	8 out of 15
4 °C	23	Low	[0–93.46]	15 out of 16
Jena University Hospital collection (n = 20)				
RT	0.5	High	[0–4747.87]	19 out of 20
RT	1	High	[2.25–1636.05]	19 out of 20
RT	2	High	[0–1336.42]	19 out of 20
RT	4	Low	[0–655.67]	12 out of 20

### Impact of extreme in vivo conditions on LacaScore

To evaluate the impact of high baseline lactic acid concentrations on the LacaScore, we collected blood from five healthy donors after a half-marathon. One of them had ingested a commercially available 2 g ascorbic acid tablet on the day preceding the half-marathon. For each donor, blood samples were collected and stored on ice or at RT for 1, 2, 3, 4, and 6 h and the quality of the sample according to the LacaScore algorithm was determined (Supplementary Data 5). The high lactic acid and ascorbic acid baseline concentrations had an impact on the LacaScore diagnostic accuracy for sample quality and fitness-for-purpose, with a reduction in accuracy (57 %).

## Discussion

Pre-analytical variations should not be neglected in any “-omics” biomarker discovery research project since the pre-analytical factors are the major source for inappropriate or inconsistent sample quality. Specimen processing should be conducted in a standardized way, especially when more than two biobanks or research institutes are involved.

Among all “-omics” analyses, metabolomics is one of the most sensitive to pre-analytical variations. Reproducibility and reliability of the analytical data can be increased by sample processing method validation and assessment of significant changes resulting from non-standardized sample handling. We have previously published an EDTA, RT/2000×*g* centrifugation plasma processing method validation (Ammerlaan et al. 2014a). EDTA plasma is the most commonly collected plasma type and also the most frequently used in metabolomics analyses. We reported that the method is reproducible, robust to centrifugation temperature and centrifugation brake conditions, with only 2–4 % of the GC–MS-detected metabolites being significantly modified under different conditions (Ammerlaan et al. 2014a). The robustness to different pre-centrifugation conditions on plasma GC–MS metabolomics was addressed in the present study.

### Impact of pre-analytical conditions on plasma metabolites

The results of this study show that the temperature at which blood samples are kept between the time of collection and the time of centrifugation and separation of the plasma from the blood cells is crucial for the plasma metabolomics data outcome. The levels of 48 out of 236 detected metabolites (20.3 %) were significantly altered when comparing blood samples stored at RT with ice-stored samples. Many standard operating procedures do not use ice for blood collection. However, for metabolomics applications, sample processing on ice is crucial and documentation of the critical pre-analytical steps in a Standard PREanalytical Code (SPREC) is recommended (Betsou et al. 2010; Bervoets et al. 2015). Two known metabolites, ascorbic acid and lactic acid, showed the highest temperature-dependent instability and we have demonstrated that their ratio can be used as quality control marker for plasma sample quality evaluation.

In humans, ascorbic acid needs to be provided in the diet and l-ascorbic acid (LAA) is oxidized to dehydroascorbic acid (DHAA) after exposure to high temperature, pH, light, oxygen or metals, and irreversible hydrolysis of DHAA leads to by-products such as oxalic acid, l-threonic acid or l-xylose (Spínola et al. 2014). A recent ascorbic acid stability study showed that ascorbic acid levels were significantly reduced in human blood plasma stored at RT compared to storage on ice, which is in accordance with the results presented in this study (Karlsen et al. 2007).

A marked loss of ascorbic acid in EDTA plasma within 30 min of blood collection was previously reported at RT (Chung et al. 2001), and another study showed plasma ascorbic acid was stable when blood was stored for up to 6 h at 4 °C (Margolis et al. 1991). EDTA vacutainers have been shown to be superior to other types of vacutainers in maintaining low ex vivo oxidation of ascorbic acid (Lykkesfeldt 2012). Degradation of ascorbic acid may be due to oxidation and/or transformation to oxalate (Wilson and Liedtke 1991). This degradation may occur in the plasma sample itself, and it has been shown that ascorbic acid is stable in samples extracted with meta-phosphoric acid and placed on a cool autosampler for at least 10 h (Kand’ar and Zakova 2008). Low temperature is a key factor in preventing the oxidation of LAA and related studies indicate stability at −80 °C (Spínola et al. 2014). However, because of potential residual enzymatic activities and instability over very long-term −80 °C storage, cryopreservation in liquid nitrogen is preferred in the scope of metabolomics analyses. This had already been suggested in 2005 by the EPIC consortium who had shown that ascorbic acid can be reliably measured in plasma samples stored in liquid nitrogen for long periods without any stabilizing agents (Jenab et al. 2005).

In contrast to decreased ascorbic acid levels, lactic acid levels were significantly higher in plasma from blood samples kept at RT compared to blood samples stored on ice. The significant changes of lactic acid can be explained by anaerobic reactions that occur over time. When collecting a sample via venepuncture, low oxygen venous blood is drawn into a vacuum container. Aerobic reactions from blood cells other than red blood cells, such as platelets and white blood cells, rapidly consume the remaining oxygen inside the collected blood sample. At RT the enzymatic reactions occur faster than at 4 °C and the oxygen depletion is much faster. This probably leads to a shift into anaerobic energy production, such as lactic acid production. Furthermore, erythrocytes, the most abundant cells present in the blood, do not have any organelles, such as mitochondria. Therefore, energy production is only performed via glycolysis, leading to lactic acid production. Lactic acid rapidly accumulates in the cells and is excreted into the plasma by either monocarboxylate transport systems or non-ionic diffusion (Simchowitz and Textor 1992; De Bruijne et al. 1983). These enzymatic reactions are slowed down at lower temperatures.

Recently, it has been shown that lactic acid levels were significantly higher in whole blood samples stored at RT for 30 min compared to whole blood samples stored on ice (Seymour et al. 2011). In similar studies, NMR measurements showed that lactic acid levels are increased in samples processed at RT for pre-centrifugation delays of 4 and 24 h. In accordance with the results obtained in this study, this increase was not observed when samples were stored on ice (Fliniaux et al. 2011). In conclusion, EDTA plasma samples are stable and fit-for-purpose for metabolomic analyses after 30 min blood pre-centrifugation delay at RT.

### LacaScore

In this study, we propose a novel plasma sample quality marker based on the ratio of ascorbic acid to lactic acid, the LacaScore. Recently, a review was published on potential quality control tools for different types of biospecimens and different types of pre-analytical variations, and ascorbic acid was identified as one of the top quality control marker candidates (Betsou et al. 2013). The reference ranges of ascorbic acid in the general population are 44.9–76.7 μM (Lentner 1981), whereas ascorbic acid concentration in plasma has been reported to be around 134 μM after oral administration of 1–2 g vitamin C (Spínola et al. 2014). The reference ranges of lactate in the general population are 740–2400 μM (Lentner 1981). Therefore, the reference range for the LacaScore (ascorbic acid to lactic acid ratio multiplied by 10^5^) is between 1871 and 10,365. Prolonged pre-centrifugation delay at RT decreases this ratio. More specifically, based on the results of this study, after 3 h at RT, the range for ascorbic acid in plasma will be 0–2 μM, while the range for lactate will be 2520–4849 μM (Supplementary Data 1). The LacaScore will therefore be between 0 and 79.37. A LacaScore lower than 5.2 indicates the blood sample has a pre-centrifugation delay of at least 3 h at RT and plasma samples unfit for metabolomics applications.

We conducted two studies to validate the LacaScore as a quality control marker. First, our method was applied to three independent plasma sample collections: a blinded dataset of 25 previously collected plasma samples (Luxembourg), giving a diagnostic accuracy of 88 %, a dataset of independently collected plasma samples from 16 donors (Geneva), giving a diagnostic accuracy of 71 % and a dataset of independently collected plasma samples from 20 donors (Jena), giving a diagnostic accuracy of 86 %. Prediction accuracy might increase with the use of a larger sample size for calculating the LacaScore decision threshold. Second, we investigated the impact of extreme ascorbic acid and lactic acid concentrations on the LacaScore diagnostic accuracy. Abnormal ascorbic acid concentrations have been shown to be associated with diseases, such as stroke and acute pancreatitis (Sanchez-Moreno et al. 2004; Scott et al. 1993). In addition, ascorbic acid supplementation greatly impacts the measured levels in blood by doubling or tripling its blood concentration (Padayatty et al. 2004). Physical exercise has not only been shown to cause high lactic acid levels but also a transient increase in circulating ascorbic acid (Peake 2003). We suspect that the LacaScore might not be applicable in cases of hyperoxalaemia (with very low ascorbic acid levels, 6.5 ± 18.6 μM) (Lentner 1981) or in cases of acute ethanolaemia (with very high lactate levels, 3500 ±  4300 μM) (Lentner 1981). It has been shown that plasma lactate levels increase by 2–12 times after running, due to production of lactate by skeletal muscles (Kondoh et al. 1992). In blood samples collected from runners after a semi-marathon, diagnostic accuracy was reduced to 57 % due to high concentrations of ascorbic acid and lactic acid. Therefore, to guarantee a proper sample quality evaluation, we recommend avoiding the use of the LacaScore with EDTA plasma samples from donors with extremely high lactic acid inducing conditions, such as intensive exercise, ethanolaemia, acute sepsis, cardiac arrest or trauma (Trzeciak et al. 2007; Kliegel et al. 2004; Abramson et al. 1993). Since both ascorbic acid and lactic acid are present and quantifiable in other types of biological fluids, namely cerebrospinal fluid and urine, use of the proposed tool might also be applicable to these types of biofluids. Concerning serum and plasma sample types, we propose use of the LacaScore with EDTA plasma. The LacaScore is not applicable to citrate plasma in which the ascorbic acid is stable over time (Trezzi, unpublished data) and may not be applicable to heparinized plasma and serum in which the ascorbic acid degradation has been shown to be slower than in EDTA plasma (Ching et al. 2002). In summary, the scope of application of the LacaScore is defined as the following: *EDTA plasma samples, ascorbic acid concentration <* *15* *μM and lactic acid concentration <* *10* *mM.* When the scope of application is defined as above, the diagnostic accuracy in the marathon runners cohort is 71 % (Supplementary Data 5), similar to that in all the other sample cohorts.

The reported LacaScore thresholds were calculated with the enzymatic assays from Abcam and Bioassay Systems. If different lactic acid and/or ascorbic acid measurement methods are used, the LacaScore thresholds may need to be re-calculated. In order to demonstrate this, we have measured the lactic acid concentrations in the Jena collection samples with the Architect method and showed that the Architect and Abcam methods are not commutable (Supplementary Data 6).

Given the high specificity of 97 %, the LacaScore is an appropriate tool for the selection of EDTA plasma samples which must be of high pre-centrifugation quality. This comes admittedly at the cost of a limited number of “false negative” samples, samples “rejected” when in fact of appropriate quality. Each biobank or research laboratory may define its acceptance criteria for the LacaScore-“indeterminate” samples. The LacaScore is also suitable for evaluating the overall quality of EDTA plasma collections. Here again, each biobank or research laboratory may define its acceptance criteria for the overall quality of a collection. Finally, the LacaScore can assist researchers and biobanks in stratifying legacy plasma samples, in the scope of downstream analyses which are known to be sensitive to the plasma pre-centrifugation conditions.

## Electronic supplementary material

Below is the link to the electronic supplementary material.

Supplementary material 1 (XLSX 16 kb)

Supplementary material 2 (XLSX 17 kb)

Supplementary material 3 (XLSX 15 kb)

Supplementary material 4 (XLSX 15 kb)

Supplementary material 5 (XLSX 35 kb)

Supplementary material 6 (XLSX 10 kb)
